# Rat Bone Marrow-Derived Schwann-Like Cells Differentiated by the Optimal Inducers Combination on Microfluidic Chip and Their Functional Performance

**DOI:** 10.1371/journal.pone.0042804

**Published:** 2012-08-03

**Authors:** Xiliang Tian, Shouyu Wang, Zhen Zhang, Decheng Lv

**Affiliations:** Department of Orthopedics, First Affiliated Hospital of Dalian Medical University, Dalian, Liaoning, People's Republic of China; Stanford University School of Medicine, United States of America

## Abstract

Numerous researches demonstrated the possibility of derivation of Schwann-like (SC-like) cells *in vitro* from bone marrow stromal cells (BMSCs). However, the concentration of the induce factors were different in those studies, especially for the critical factors forskolin (FSK) and β-heregulin (HRG). Here, we used a new and useful method to build an integrated microfluidic chip for rapid analyses of the optimal combination between the induce factors FSK and HRG. The microfluidic device was mainly composed of an upstream concentration gradient generator (CGG) and a downstream cell culture module. Rat BMSCs were cultured in the cell chambers for 11 days at the different concentrations of induce factors generated by CGG. The result of immunofluorescence staining on-chip showed that the group of 4.00 µM FSK and 250.00 ng/ml HRG presented an optimal effect to promote the derivation of SC-like cells. Moreover, the optimal SC-like cells obtained on-chip were further tested using DRG co-culture and ELISA to detect their functional performance. Our findings demonstrate that SC-like cells could be obtained with high efficiency and functional performance in the optimal inducers combination.

## Introduction

Cell-based therapy is a promising strategy for the treatment of peripheral nerve injury [1]–[3]. Schwann cells (SCs) are recognized as the most important seed cells for nerve tissue engineered grafts. They are essential for the formation and maintenance of the myelin sheath around axons after nerve injuries as they produce various neurotrophic factors and extracellular matrix to guide and promote axonal growth [4]–[6]. However, cultured autogenic SCs have limited clinical application because of the concomitant donor site morbidity and the slow growth of these cells *in vitro* [7], [8]. More recently, introduction of bone marrow-derived Schwann-like cells into nerve graft scaffolds has achieved promising functional recovery for peripheral nerve repair [9], [10].

Several researches demonstrated the possibility of derivation of SC-like cells from BMSCs *in vitro*. The phenotype of the differentiated BMSCs resembled that of Schwann cells [9], [11]–[14]. It is now clear that the sequential administration of various factors β-mercaptoethanol (β-ME), all-trans-retinoic acid (RA), followed by a cocktail of forskolin (FSK), platelet derived growth factor-AA (PDGF-AA), basic fibroblast growth factor (bFGF), and β-heregulin (HRG) effectively induce the differentiation of BMSCs into myelinating cells, thus assisting the regeneration of nerve fibers. Among these induce factors, FSK and HRG are recognized as the most important ones for BMSCs differentiating into myelinating cells. However, the concentration of these induce factors were different in different studies, especially for the critical factors FSK and HRG [3], [9], [15], [16]. The optimal concentrations of stimulating factors are critical for the commitment of stem cells and the ideal transplanted SC-like cells should be induced with high performance and proliferate rapidly. Therefore, it is necessary to develop a new and useful methods to investigate the optimal combination of induce factors for BMSCs differentiating into SC-like cells.

Microfluidic technology has proven very useful for high throughput analyses at cellular level and was widely applied on cell-based research. On-chip-based microfluidic devices could be used for microscale cultures, creating specific cellular microenvironments as well as analysis and manipulation of biological samples [17]–[19]. In this respect, the microfluidic gradient chip is a powerful tool for studying the effects of different concentrations of stimulating factors compared with a traditional cell culture system.

In this study, we investigate the optimal combination between the inducers FSK and HRG using a new method of integrated microfluidic gradient device. Moreover, the ideal SC-like cells differentiated in the optimal combination of induce factors were co-cultured with DRG neurons for assessment of the bioactivity, and detected the expressions of neurotrophins BDNF, NGF and NT-3 using enzyme-linked immunosorbent assay (ELISA).

## Results

### Design of microfluidic device

**Figure 1:**
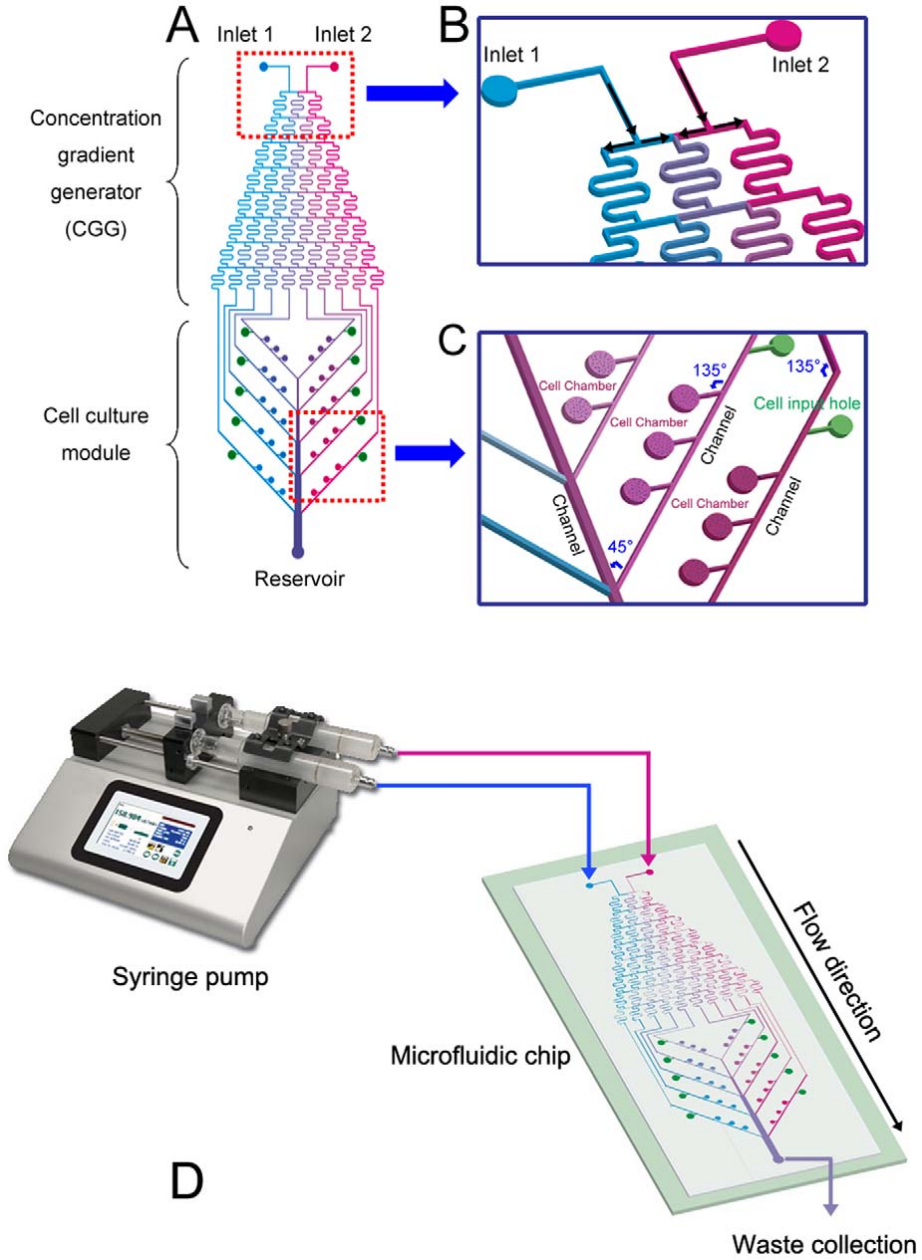
Microfluidic device design. (A) Layout of the integrated microfluidic device mainly composed of an upstream CGG and a downstream cell culture module. (B) The microfluidic channels in the upstream dilution module generate several concentrations of the stimulus by continuous flow diffusive mixing of adjacent laminar flow streams in the serpentine channels. (C) Three chambers were connected to every channel of downstream culture module. Solutions would diffuse into the chambers by osmosis till the concentration of the mixture in the chambers is uniform to the connected channel. (D) Microfluidic device operation. A syringe pump was connected to a microfluidic chip by teflon tubes. It drived the solutions into the chip at a flow speed of 0.1 ml/min continuously in laminar mode, meanwhile, waste liquid was collected.

The microfluidic device used in this work was designed by AutoCAD software (Autodesk, USA), which have an upstream concentration gradient generator (CGG) and a downstream cell culture module (Fig. 1A). The design of the CGG was based on the work previously presented by Jeon *et al*. [20]. The dimensions of the microfluidic channel were 200 µm (width)×200 µm (height). The microfluidic channels in CGG generate several concentrations of the stimulus by continuous flow diffusive mixing of adjacent laminar flow streams in the serpentine channels (Fig. 1B). The cell culture module was composed of an array of round cell culture chambers that are integrated with the microfluidic channels extended from the CGG unit. The channels were 45°beveled, and three chambers were connected to channels in the inside for performance of three independent cell groups. The cell chambers were 700 µm in diameter and 200 µm in height. The volume of each chamber is around 0.1 µL. Upstream of each channel was connected to a cell input hole in the opposite side. As the microfluidic channels were syntropic 45°angle to the chamber, cells flowing through the channels would partly get into the chambers or partly continue to flow through the channels and get into the downstream chambers until they were filled (Fig. 1C). Furthermore, the extracellular matrix could be seeded into the chambers through the input holes and channels. Teflon tubes with outer diameter slightly larger than the inner diameter of the inlet or outlet ports were inserted into the holes to make the fluidic connections. The pieces of tubes were then connected to a syringe pump to complete the fluidic device (Fig. 1D).

For the current device, if one solution (concentration 0) and the other one (concentration C) were introduced into CGG, the concentrations interval from channel 1^st^ to 10^th^ are, respectively, 0, 1/9C, 2/9C, 3/9C, 4/9C, 5/9C, 6/9C, 7/9C, 8/9C and C according to the equation described by Jeon *et al*. [20]. When solutions flow down through each channel, stimulating factors and other substance would diffuse into the chambers by osmosis till the concentration of the mixture in the chambers is uniform and consistent with the connected channels.

### Identification of BMSCs

**Figure 2:**
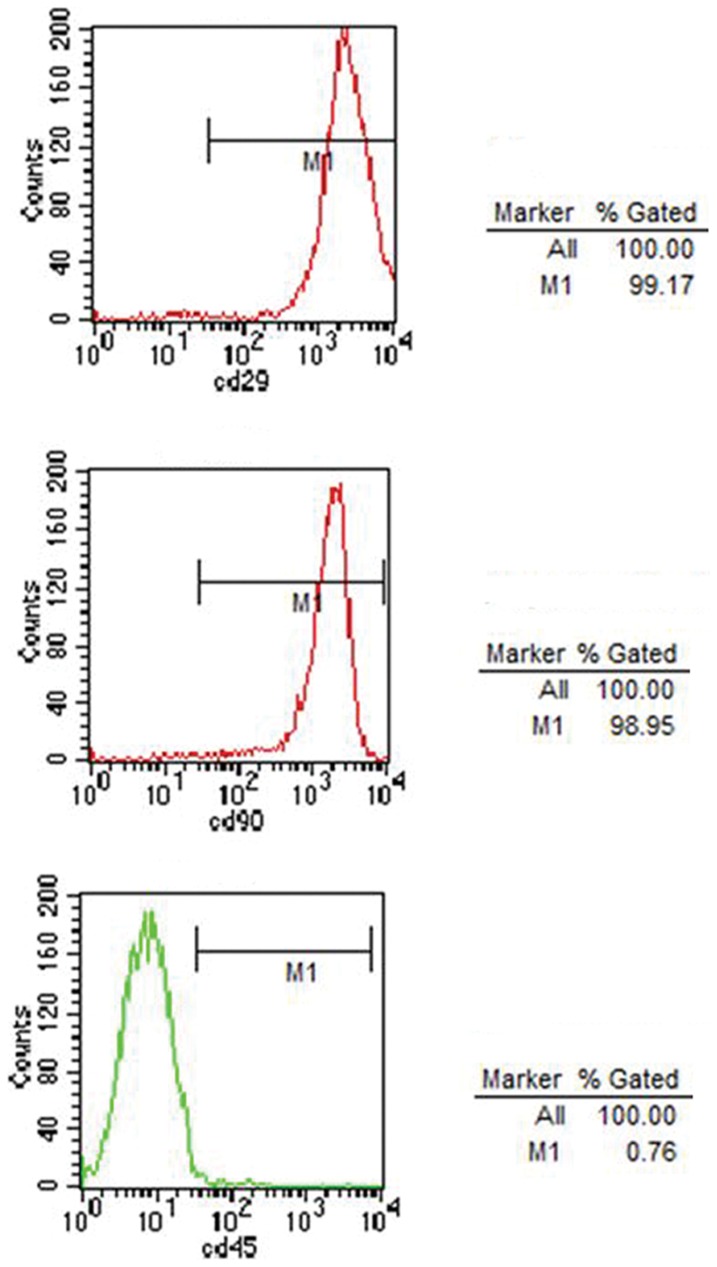
FACS analysis for BMSCs surface markers. The cells were indicated positive for CD29 (99.17%) and CD90 (98.95%), but negative for CD45 (0.76%) after P3.

**Figure 3:**
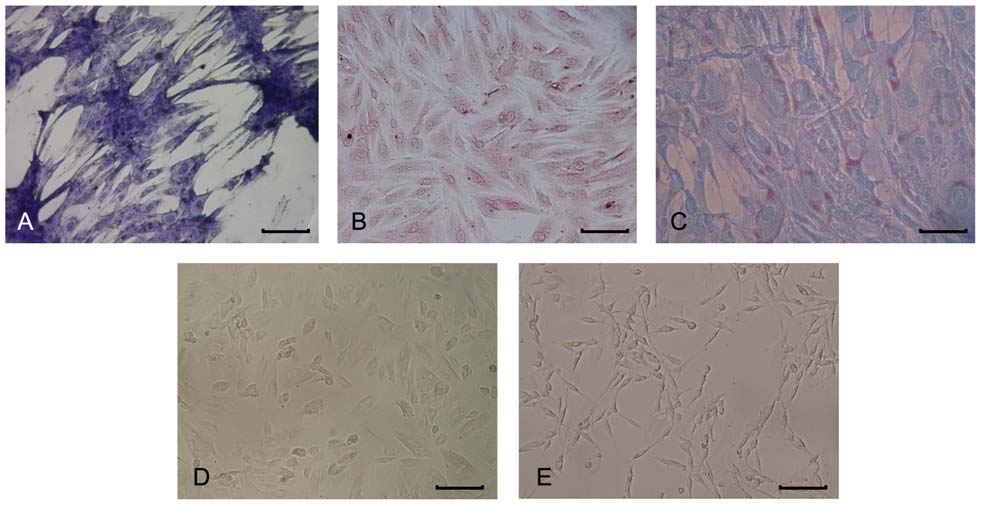
Multilineage potential and morphological change of BMSCs after differentiation. BMSCs (P3) differentiated into chondrocytes (Toluidine blue staining: A), osteocytes (Alizarin red staining: B) and adipocytes (Oil Red staining: C). (D) Undifferentiated BMSCs showed a flat, fibroblast-like morphology. (E) After differentiating into SC-like cells, the cells changed to a bipolar, spindle-like shape. Scale bar: 50 µm.

Phase-contrast microscopy revealed that BMSCs (passage 0–3, P0–P3) formed densely packed fibroblast-like colonies adherent on tissue culture plastic. Mouse-anti rat CD29, CD45 and CD90 antibodies were used to detect antigenic markers on the surfaces of BMSCs. Flow cytometry analysis demonstrated that the cells obtained were uniformly positive for CD29 (99.17%) and CD90 (98.95%), but negative for CD45 (0.76%) after P3 (Fig. 2). The multipotency of BMSCs (P3) was demonstrated by their ability to differentiate into chondrocytes, osteoblasts and adipocytes in appropriate culture conditions. The sulphated proteoglycan of chrondrocytes was stained blue with Toluidine Blue (Fig. 3A), areas of calcification around osteoblasts were labelled red with Alizarin red (Fig. 3B) and the lipid droplets in the adipocytes were stained red with Oil Red (Fig. 3C).

### Immunocytochemistry for SCs markers in microfluidic chip

**Figure 4:**
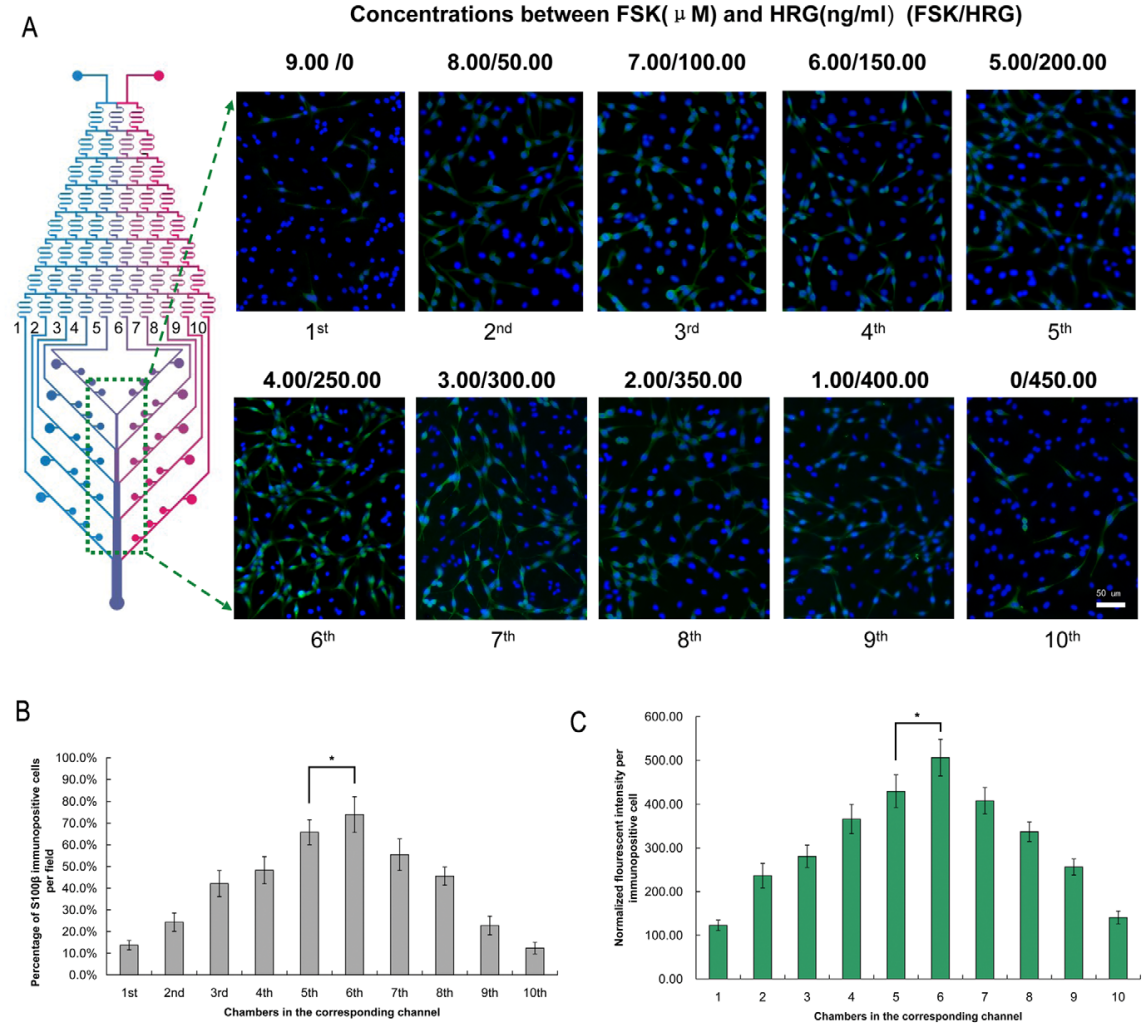
Immunocytochemistry for S100β immunopositive cells on-chip. (A) The cells in every chamber were stained with S100β antibody (green) and counterstained with DAPI (blue). Scale bar: 50 µm. (B) Among the combinations of the induce factors, the 6^th^ group (4.00 µM FSK and 250.00 ng/ml HRG) indicated the largest number of S100β immunopositive cells. Control vs. the 5^th^ group (5.00 µM FSK and 200.00 ng/ml HRG): *p<0.05. (C) The average expression of S100β in per immunopositive cell reflected by normalized fluorescent intensities was at the highest level in the same group. Control vs. the 5^th^ group: *p<0.05. The normalized fluorescent intensities per cell were determined by the total fluorescent intensity in a field divided by the number of immunopositive cells in that field. All the experiments were repeated at least three times.

**Figure 5:**
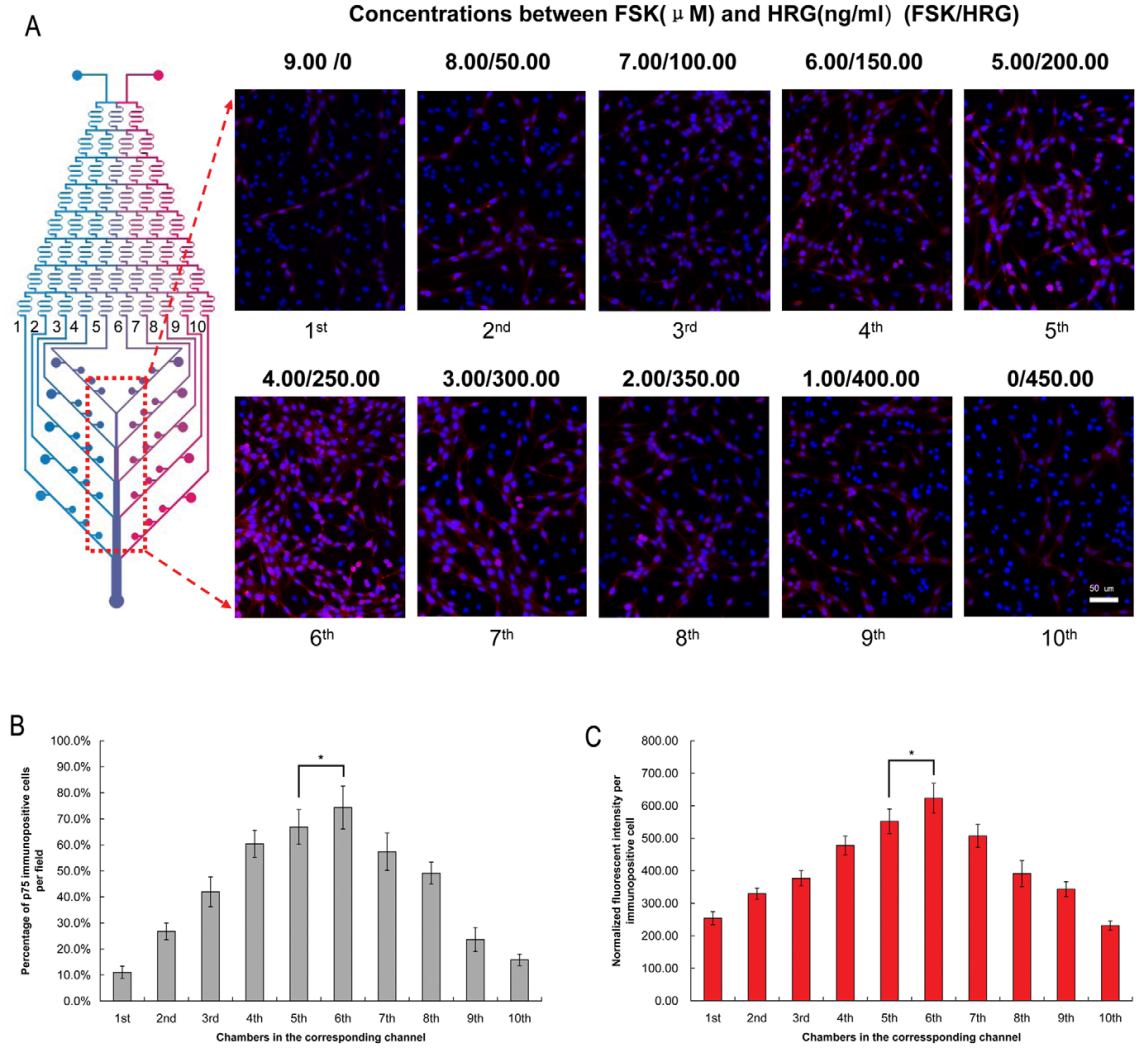
Immunocytochemistry for p75^NTR^ immunopositive cells on-chip. (A) Cells cultured in chambers were stained with p75^NTR^ antibody (red) and counterstained with DAPI (blue). Scale bar: 50 µm. (B) In consistent with S100β expression, cells cultured in the same combination of induces factors showed the largest number of p75^NTR^ immunopositive cells. Control vs. the 5^th^ group: *p<0.05. (C) The normalized fluorescent intensity per immunopositive cell was at the maximal gray scale in the same group. Control vs. the 5^th^ group: *p<0.05. All the experiments were repeated at least three times.

11 days after differentiation on-chip, the BMSCs changed from a monolayer of large, flat cells (Fig. 3D) to a bipolar, spindle-like shape (Fig. 3E). Immunocytochemical analyses were performed on-chip to show the SCs markers S100β and p75^NTR^. Clearly, cells in the chambers extended from the 6^th^ channel (the combination between FSK and HRG was 4.00 µM and 250.00 ng/ml) indicated the largest number of immunopositive cells and the average expression of S100β and p75^NTR^ in per immunopositive cell reflected by normalized fluorescent intensities was at the highest level in the same group (Fig. 4 and Fig. 5). The normalized fluorescent intensities per cell were determined by the total fluorescent intensity in a field divided by the number of immunopositive cells in that field. Therefore, the group of 4.00 µM FSK, 250.00 ng/ml HRG, 5.00 ng/ml PDGF-AA and 10.00 ng/ml bFGF was recognized as the optimal combination of the induce factors.

### DRG co-culture functional bioassay

**Figure 6:**
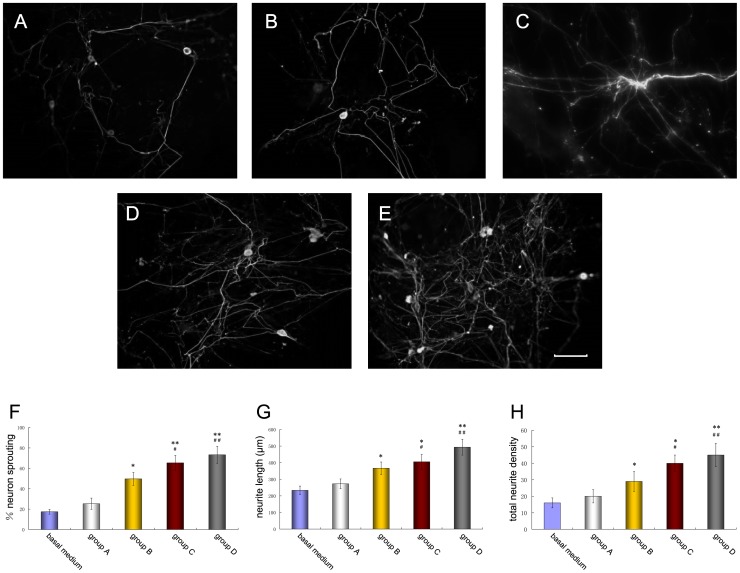
DRG neurons co-cultured with cells of the four groups. (A–E) Immunocytochemical staining for βIII-tubulin (FITC) to show neurites sprouting from DRG neurons after co-culture for 24 h with basal media (control, A), undifferentiated BMSCs (B), SC-like cells cultured in conventional differentiate media (C), SC-like cells cultured in optimal differentiate media (D) and Schwann cells (E). Scale bar: 100 µm. (F–H) Three independent parameters measured: percentage of DRG neurons sprouting neurites (F), length of longest neurite (G) and total neurite density (H). Data were listed as mean ± S.E.M. Control vs. group A and basal media: *p<0.05, **p<0.01; Control vs. group B: ^#^ *p*<0.05, ^##^ *p*<0.01.

Immunocytochemistry for βIII-tubulin showed the extent of neurite outgrowth for DRG neurons co-cultured with basal media alone and cells in group A (undifferentiated BMSCs), group B (SC-like cells cultured in conventional differentiate media), group C (SC-like cells cultured in optimal differentiate media) and group D (Schwann cells) (Fig. 6A–E).

These results were confirmed with quantification, which showed a significant increase in the neurite sprouting, length of the longest neurite and neurite density when DRG neurons were co-cultured with cells of group C as compared with control basal media (p<0.01) and cells of group A (p<0.01) (Fig. 6F–H). DRG neurons co-cultured with cells of group B showed some neurite outgrowth, which did not seem to be as abundant as co-culture with cells of group C and D (p<0.05). Cells of group C promoted the more neurite outgrowth similar to SCs (group D).

### Secretion of bioactive neurotrophins

**Figure 7:**
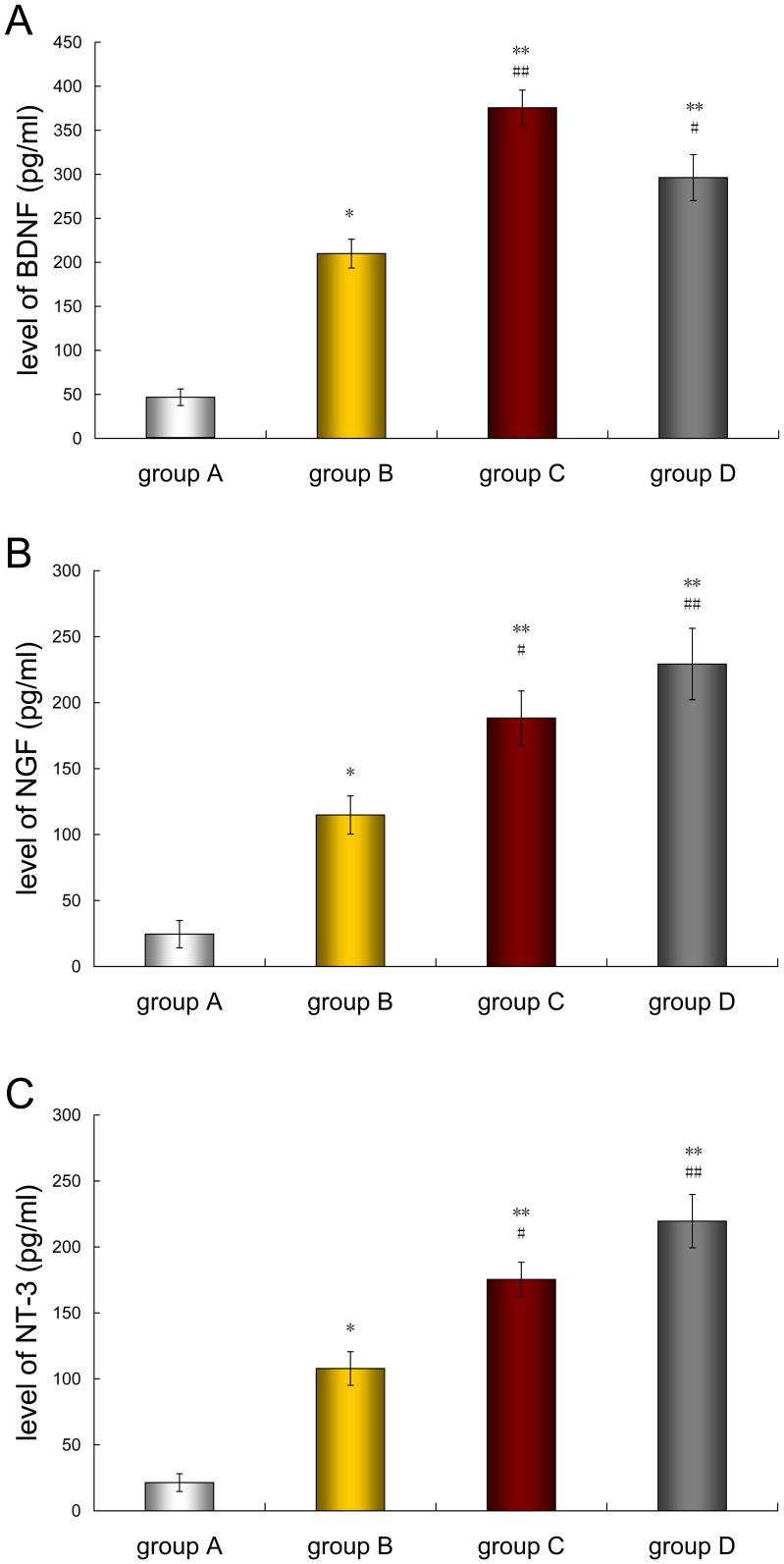
Assessment of neurotrophins production. ELISA quantification of BDNF (A), NGF (B) and NT-3 (C) secreted by the cells of the four groups. Data were listed as mean ± S.E.M. Control vs. group A: *p<0.05, **p<0.01; Control vs. group B: ^#^ *p*<0.05, ^##^ *p*<0.01.

ELISA quantification indicated the secretion level of BDNF, NGF or NT-3 in the mediums of group A, B, C and D. These neurotrophins secreted by cells in group C were at significantly higher levels than that in group A and B, and the levels were similar to SCs (group D) (Fig. 7).

## Discussion

### Differentiation of BMSCs into SC-like cells

Recently, cell-based therapy is a promising strategy for the treatment of peripheral nerve injury. Schwann cells display a number of characteristics that suggest their role in facilitating peripheral nerve regeneration [21], [22], but the limited Schwann cell resource and the xenogeneic graft immunorejection reaction hindered its further application. BMSCs are an attractive alternative candidate because they exhibit several important and potential advantageous features both in PNS and CNS regeneration. They can be obtained easily, and display an unorthodox plasticity. BMSCs can take on several cell phenotypes after being induced *in vitro* by different inducer factors. However, many studies have speculated that the number of mesenchymal stem cells within the bone marrow and their differentiation capacity declines with age [23], [24]. Therefore, it is essential for the differentiation of BMSCs as soon as possible.

Classical sequential administration of β-ME, RA and a mixture of FSK, bFGF, PDGF and HRG can induce BMSCs differentiating into cells with a spindle-like shape that express S100β and p75^NTR^ similar to genuine SCs [9], [11]–[14]. β-ME and RA are considered to work as triggering factors, which change the morphological and transcriptional characteristics of BMSCs [25], [26]. bFGF and PDGF function as the mitogen for mesenchymal cells [27]. HRG, a subtype of neuregulin, instructively influences the decision of cell fates and could induce neural crest cells to develop selectively into Schwann cells [28]. FSK increases the level of intracellular cyclic adenosine monophosphate (cAMP), which increases mitogenic responses by stimulating the expression of growth factor receptors. As cAMP elevation was reported to enhance the responsiveness of cells to trophic factors, FSK together with bFGF, PDGF and HRG could have a complementary effect in enhancements of these factors to BMSCs [29]. However, the concentration of these induce factors are different in several studies, especially for the critical factors FSK and HRG [3], [9], [15], [16]. The optimal concentrations of stimulating factors are critical for the commitment of stem cells and the ideal transplanted SC-like cells should be induced with high performance and proliferate rapidly. Therefore, it is necessary to investigate the optimal combination of induce factors for BMSCs differentiating into SC-like cells. Here, we obtained the optimal combination of inducers concentration using the integrated microfluidic gradient chip, and meanwhile avoided a large number of repeated tests. The results will be very valuable in future experiments.

### Microfluidic device for analyses at cellular level

In this work, we presented an application of microfluidic device in which a linear concentration gradient was generated via a chemical gradient generator. Such a microfluidic system can have the following advantages for BMSCs differentiation: (1) The device allows a long-term maintenance of a stable growth factor concentration gradient with low consumption of costly reagents; (2) The creation and control of concentration gradients can be freely manipulated; (3) Dimensions of the microchannels are small enough so that diffusion occurs from minutes to seconds, resulting in reducing the waiting time for a gradient to be established; (4) Real-time cell observation and cellular assays in the microfluidic network could be operated easily and efficiently; (5) The entire device is simple, small and portable so that the microfluidic system can be a component of other biological experimental systems, and (6) it could provide reproducible and identical experimental conditions for repetitive tests.

In the previous studies, the effect of induce factors on BMSCs differentiation was completed using a wide gap of induce factor concentration (i.e. 0, 50, and 100 ng/ml) in a monolayer culture. It is actually impossible for researchers to perform many parallel experiments using conventional methods to figure out the optimal condition with limited grouping. In comparison, our platform was able to generate detailed concentrations of induce factors in a rapid and automated pattern. In this study, we introduced the combined concentration of 9.00 µM FSK and 450.00 ng/ml HRG to the microfluidic device. The concentration gradient of FSK (from channel 1^st^ to 10^th^) was 9.00, 8.00, 7.00, 6.00, 5.00, 4.00, 3.00, 2.00, 1.00 and 0 µM, and the concentration gradient of HRG (from channel 1^st^ to 10^th^) was 0, 50.00, 100.00, 150.00, 200.00, 250.00, 300.00, 350.00, 400.00 and 450.00 ng/ml with an decrease or increase by 1/9 of highest concentration, respectively.

The morphological changes of BMSCs were clearly evident within 4–5 days of culture in the presence of induce factors. After induction along a glial lineage for 12 days, the differentiated cells displayed the bipolar, elongated spindle-shape, which is characteristic of SC spindle-like morphology. The whole processes from cell culture to the analyses of protein expression were done continuously and conveniently without time wasting.

### Functional performance of the optimized SC-like cells

In order to further confirm that BMSCs differentiated in optimal induce media display the more functional characteristics similar to SCs, we established a DRG co-culture system to assess the functional performance. The result revealed that SC-like cells cultured in optimal differentiate media could significantly promote more neurite outgrowth, elongation and branching as compared with undifferentiated BMSCs and SC-like cells cultured in conventional differentiate media, and behaved similar to SCs. We supposed that the increase in neurite outgrowth was the result of the up-regulated secretion of neurotrophic factors, such as BDNF, NGF and NT-3. Further assessment by ELISA demonstrated that SC-like cells cultured in optimal differentiate media highly expressed the above neurotrophins.

Meaningfully, we have found that the secretion level of BDNF by SC-like cells cultured in optimal induce media was higher than that by SCs, which is consistent with the results Mahay *et al*. [30] and Peng *et al*. [2] have previously reported. BDNF has been reported to have a regulation in axon myelination and regeneration, neuronal survival, axonal outgrowth, differentiation and glial cell proliferation [31]–[33]. BDNF is initially elevated before myelination and has been shown to modulated neuron-glia interactions in PNS and CNS [34], [35]. We propose that this increase in neurite outgrowth is mainly due to the up-regulated secretion of neurotrophin BDNF.

In summary, we originally obtained the optimal compatibility concentration of the induce factors for BMSCs differentiating into SC-like cells based on the microfluidic device. The microfluidic device we proposed is featured with parallel gradient generating network, which offers an opportunity to integrate the whole procedure of cell-based research, including cell seeding, cell culture, concentration gradients generation, induce factors stimulation, cellular morphological change monitoring and cell staining. The microfluidic-based platform enables the multiparametric analyses with simplified operation and minimum reagents consumption, thus, holds promising potential for the high-throughput screening at the cellular level.

## Materials and Methods

### Ethics Statement

The animal experiments were in accordance with the NIH Guide for the care and use of laboratory animals, and approved by the Committee on Research Animal Care of Dalian Medical University, Dalian, China.

### Fabrication of microfluidic device

The microfluidic device was fabricated using poly (dimethylsiloxane) (PDMS; Sylgard 184, Dow Corning, USA) and glass slide via soft lithography methods and rapid prototyping techniques [36], [37]. Briefly, a film mask containing the channel design (1∶1 printed) was used in contact photolithography with SU-8 photoresist (Microchem, USA) to generate a negative mold (200 µm thick) on a cleaned silicon wafer. Positive replicas with embossed channels were fabricated by molding PDMS (2 mm thick) against the SU-8 master mold. Then the PDMS replica was cut down and drilled holes with 3 mm inner diameter using a sharpened needle at inlets and outlets. Finally, the side of the PDMS slab with embossed channels was irreversibly bonded to a 10 cm×5 cm glass slide assisted by oxygen plasma surface treatment (150 m torr, 50 W, 20 s).

### BMSCs culture and characterization

Rat BMSCs were isolated as described by previous studies [7], [10]. Briefly, femurs and tibias were dissected from young adult Sprague Dawley (SD) rats (80–120 g). Following epiphyseal puncture, marrow cells were flushed out and cultivated in media comprising DMEM plus 10% (v/v) FBS and 1% (v/v) penicillin-streptomycin (all Invitrogen, USA) at a density of 2×10^5^/cm^2^ at 37°C,in 95% humidified air and 5% CO_2_ (P0). The isolation of BMSCs was based upon their ability to adhere to plastic surfaces. After 72 h, non-adherent cells were removed and remaining cells were maintained in culture for another 8–10 days with media refreshed every 3 days. At 80–90% confluence, the cells were detached by 0.25% trypsin containing 0.1% EDTA (Invitrogen) and re-seeded in a new culture flask at a density of 1×10^4^ cells/cm^2^. Cells at P3–P5 were used in later experiments.

To confirm the multipotency of the BMSCs, the cultures at P2 were treated with different induction media for 3 weeks [2], [6]. Osteogenic induction media comprised 10% FBS, 10 mM β-glycerophosphate, 0.1 µM dexamethasone and 100 µg/ml ascorbate (all Sigma-Aldrich, USA) in DMEM (Invitrogen). Chondrogenic induction media was DMEM supplemented with 10 nM dexamethasone, 50 µg/ml ascorbate, 40 µg/ml proline, 10 ng/ml transforming growth factor β1 (all Sigma-Aldrich) and 1% insulin-transferrin-selenium (ITS-Plus; BD Falcon, USA). Adipogenic induction media contained 10% FBS, 1 µM dexamethasone, 10 µg/ml insulin, 0.5 mM 3-isobutyl-1-methylxantine and 200 µg/ml indomethacin (all Sigma-Aldrich) in DMEM. When the differentiation processes were completed, the cultures were fixed with 4% paraformaldehyde in 0.01 M PBS (pH 7.4) and stained for osteoblasts, chondrocytes and adipocytes using Alizarin red, Toluidine Blue and Oil Red (all Sigma-Aldrich) respectively.

The BMSCs were additionally identified by flow cytometry. After detachment from the culture flasks, single-cell suspensions were washed three times in PBS. 10^6^ cells were used per sample and incubated with mouse-anti rat CD29, CD90 and CD45 antibodies (all BioLegend, USA) conjugated with fluorescein isothiacyanate (FITC) or phycoerythrin (PE) separately in the dark for 20 min on ice. Cells were washed once and re-suspended in 1 ml PBS, and then examined by FACScan.

### BMSCs seeding, culture and differentiation on the chip

Prior to cell seeding on the microchip, microchannels were sterilized with 75% (v/v) ethanol for 2 h and then dried at room temperature in a clear hood. The cell culture chambers were filled with poly-l-lysine solution (0.01%, m/v) (Sigma-Aldrich) for 1 h in order to coat its inner surface. BMSCs (P3) cultured in the dishes were digested with 0.25% trypsin and resuspended in glutamine-free α-MEM (Hyclone, USA), and then they were injected into the culture chambers at 5×10^6^ cells/ml *via* the downstream cell input holes by syringe pump. The total injected volume was 20 µL. Because the PDMS is flexible, the exports of CGG were pinched using a clamp before injecting the cell suspension into culture chambers. This operation can prevent the cell suspension from flowing backward into the CGG. After the chambers were filled with cells, PBS buffer was slowly induced into the device to flush out the residual cells in the channels.

Cells were firstly preinduced in α-MEM containing 1 mM β-ME (Sigma-Aldrich) for 24 h (37°C, 5% CO_2_). Then the media was removed, washed with PBS for 3×5 min, and replaced with new media consisting of α-MEM, 10% FBS, and 35 ng/ml RA (Sigma-Aldrich) for 72 h (37°C, 5% CO_2_). All of the above reagents were simultaneously infused from the two inlets of CGG by syringe pump. The flow speed was controlled at 0.1 ml/min. After washed with PBS for 3×5 min, the cocktail induce factors were introduced into the microchannels. The media containing 10% FBS, 9.00 µM FSK, 5.00 ng/ml PDGF-AA, 10.00 ng/ml bFGF (absence of HRG) and another media containing 10% FBS, 450.00 ng/ml HRG, 5.00 ng/ml PDGF-AA, 10.00 ng/ml bFGF (absence of FSK) were simultaneously infused into the CGG from inlet 1 and 2. The fluid streams were repeatedly split, mixed, and recombined when traveled through the network. The concentration gradient of induce factors was established at ten outlets of CGG 30 s later. Solutions carrying different concentrations of induce factors diffused into cell culture chambers when they flow into each channel of the cell culture module. The concentrations between FSK and HRG in the cell chambers integrated with ten channels were list in Table 1. The device was then kept in an incubator at 37°C in 95% humidified air and 5% CO_2_. Cell morphologies were monitored by a phase contrast microscope (TE 300; Nikon Co., Japan) equipped with a digital camera. After incubated for 7 days, immunoreactivities for the SCs markers were tested on the chip, and immunopositive cells at this stage were referred to as SC-like cells.

**Table 1:** The concentrations between FSK and HRG in the cell chambers integrated with ten channels.

Channel No.	1^st^	2^nd^	3^rd^	4^th^	5^th^	6^th^	7^th^	8^th^	9^th^	10^th^
FSK (µM)	9.00	8.00	7.00	6.00	5.00	4.00	3.00	2.00	1.00	0
HRG (ng/ml)	0	50.00	100.00	150.00	200.00	250.00	300.00	350.00	400.00	450.00

### Immunocytochemistry for SC markers in microfluidic chip

After culture of BMSCs-derived SC-like cells inside microfluidic device, cells were fixed in cold 4% (v/v) paraformaldehyde for 20 min at room temperature. After permeabilization and blocking with 0.3% (v/v) Triton X-100 and 0.2% (w/v) bovine serum albumin for 1 h, cells were respectively probed with rabbit-anti rat S100β antibody (1∶500) and rabbit-anti rat p75^NTR^ antibody (1∶500) for overnight at 4°C. After incubation with primary antibodies, cells were washed and probed with FITC-anti-rabbit antibody (1∶200) and TRITC-anti-rabbit antibody respectively (1∶200) (All Sigma-Aldrich) as secondary antibodies for incubation at 37°C for 60 min in the dark. Finally, nuclear staining was performed by DAPI (KeyGEN, China) for 10 min. Between each step, the samples were washed with PBS buffer for 3×5 min. Fluorescent images were captured using a fluorescence microscope (Ti; Nikon Co., Japan) and analyzed using the Image-Pro Plus Imaging software (version 6.0; Media Cybernetics, USA). The experiment was repeated three times. The chamber, reflecting the individual concentration of induce factors, which showed the most immunopositive cells and expressed the maximal gray scale of S100β and p75^NTR^ was recognized with the optimal combination.

### Dorsal root ganglia (DRG) neuron harvest

Dissociated DRG neurons were harvested from 8-week-old male Sprague-Dawley (SD) rats as described previously [2]. Briefly, the spines were removed after the rats were anesthetized by peritoneal injection of sodium pentobarbital (5 mg/100 g). DRGs were isolated from the spinal cords and the epineuriums of DRGs were carefully isolated under a dissecting microscope. After dissected into pieces of about 1 mm^3^ size,the DRG were then collected into a Ham's F12 media containing 0.125% collagenase Type II for 30 min at 37°C. Collagenase was then inactivated using 33% (w/v) FBS and DRGs were dissociated by gentle trituration using a glass pipette, passed through a filter of 70 µm pore size filter (BD Falcon, UK). After centrifuging at 1000 rpm for 5 min and removing the supernatant, the dissociated DRG neurons were diluted in F12 media containing 1% N2 supplements, 1 mg/ml bovine serum albumin, and 10 µM cytosine arabinoside. The endogenous Schwann cells which naturally embedded in the isolated DRG tissue were removed after epineuriums isolation, Enzyme digestion and cytosine arabinoside inhibition. DRG neurons were seeded onto glass cover slips in 6-well plates and allowed to attach for 24 h (37°C, 5% CO_2_).

### DRG co-culture functional bioassay

DRG co-culture was established to detect the neurite outgrowth properties of undifferentiated BMSCs (group A), SC-like cells cultured in conventional differentiate media (group B), SC-like cells cultured in optimal differentiate media (group C) and Schwann cells (group D). The conventional induce factors composition for SC-like cells culture was 1 mM β-ME, 35 ng/ml RA, followed by a cocktail of 5.00 µM FSK, 200.00 ng/ml HRG, 5.00 ng/ml PDGF-AA and 10.00 ng/ml bFGF [9], [12]. Rat SCs were purchased from the Type Culture Collection of the Chinese Academy of Sciences and used as positive control for DRG co-culture and ELISA.

24 h prior to DRG neurons harvest, cells of group A, B, C and D were seeded on 1.0 µm pore size cell inserts (BD Falcon) at a density of 1.5×10^5^ cells in 2 ml/insert in 6-well plates and incubated for 48 h (37°C, 5% CO_2_). The inserts were checked for adherence and placed in wells containing DRG neurons to establish the co-culture for 24 h (37°C, 5% CO_2_). Additional wells with the cell-free inserts (basal media) were used as a control.

To evaluate the neurite outgrowth of the DRG neurons, the 6-well-plate coverslips were fixed in 4% paraformaldehyde for 30 min and immunostained with rabbit-anti rat βIII tubulin antibody (1∶1000; Sigma-Aldrich) then secondary FITC-anti-rabbit antibody (1∶200; Sigma-Aldrich). Neurite outgrowth from DRG neurons were captured by fluorescence microscopy, and quantified at 10× magnification by use of Image-Pro Plus Imaging software. Neurite outgrowth was assessed using three independent parameters: percentage of process-bearing neurons, length of longest neurite and total neurite density [38]. Three independent co-culture experiments were carried out.

### Assessment of neurotrophins production

To obtain conditioned media, 10^5^ cells of group A, B, C and D in 2 ml were seeded on 6-well plate. After incubating overnight at 37°C in 95% humidified air and 5% CO_2_, the conditioned media were collected, filtered through a 0.22 µm filter (Pall, USA), and analyzed by ELISA using rat BDNF, NGF or NT-3 sandwich ELISA kits (Chemicon, USA) according to the manufacturer's protocol. All samples were analyzed in triplicate, and the absorbance was measured at 450 nm (Multiskan MK3 mirco-plate reader; Thermo, USA).

### Statistical analysis

Statistical analysis was performed using SPSS 13.0 software (SPSS® Inc., USA). All data were expressed as mean ± S.E.M.. One-way analysis of variance (ANOVA) with Tukey's post hoc test was used to determine significant differences among groups. Statistical significance was determined as ^*^ *p*<0.05 or ^**^ *p*<0.001.
